# World Health Organization grading classification for pancreatic neuroendocrine neoplasms: a comprehensive analysis from a large Chinese institution

**DOI:** 10.1186/s12885-020-07356-5

**Published:** 2020-09-22

**Authors:** Min Yang, Lin Zeng, Neng-wen Ke, Chun-lu Tan, Bo-le Tian, Xu-bao Liu, Bo Xiang, Yi Zhang

**Affiliations:** 1 Department of Pediatric Surgery, West China Hospital of Sichuan University, Chengdu, Sichuan Province The People’s Republic of China; 2 President & Dean’s Office, West China Hospital of Sichuan University, Chengdu, Sichuan Province The People’s Republic of China; 3 Department of Pancreatic Surgery, West China Hospital of Sichuan University, Chengdu, Sichuan Province, the People’s Republic of China, No.37, Guoxue Road, Wuhou District, Chengdu, Sichuan Province The People’s Republic of China

**Keywords:** PANCREATIC neuroendocrine neoplasms, WHO, Grading, AJCC, Staging, Prognosis

## Abstract

**Background:**

Pancreatic neuroendocrine neoplasms (p-NENs) are a group of highly heterogeneous tumors with distinct clinicopathological features and long-term prognosis. In 2017, in order to better stratify patients into prognostic groups and predicting their outcomes, World Health Organization (WHO) officially updated its grading system for p-NENs which distinguished these neoplasms among Grading 1 (G1) pancreatic neuroendocrine tumors (p-NETs), G2 p-NETs, G3 p-NETs and G3 pancreatic neuroendocrine carcinomas (p-NECs). However, this new grading classification for p-NENs has not yet been rigorously validated.

**Methods:**

Data of patients who were surgically treated and histopathologically diagnosed as p-NENs at West China Hospital of Sichuan University from January 2002 to December 2018 were retrospectively collected and analyzed according the novel WHO 2017 grading classification.

**Results:**

We eventually enrolled 480 eligible patients with p-NENs in our present study, in which 150 patients with WHO 2017 G1 p-NETs, 158 with G2 p-NETs, 64 with G3 p-NETs and 108 with G3 p-NECs were identified. The estimated 5-year overall survival for patients with G1 p-NETs, G2 p-NETs, G3 p-NETs and G3 p-NECs was 75.8, 58.4, 35.1 and 11.1%, with a median survival time of 85.3mons, 67.4mons, 51.3mons and 26.8mons, respectively. Patients with G2 p-NETs present notably worse survival than those with G1 p-NETs (*P* = 0.03). Survival of G3 p-NETs were significantly worse than that of G1 p-NETs or G2 p-NETs (*P* < 0.001, *P* = 0.023, respectively), as well as that when comparing G3 p-NECs with G1 p-NETs or G2 p-NETs (*P* < 0.001, *P* < 0.001, respectively). Patients with G3 p-NECs showed statistically shorter survival than those with G3 p-NETs (*P* < 0.001). Both WHO 2017 and 2010 grading criteria could be independent predictor for the OS of p-NENs (*P* = 0.016, *P* = 0.022; respectively). The 95% confidence intervals of WHO 2017 grading classification (0.983–9.454) was slightly smaller than that of WHO 2010 criteria (0.201–13.374), indicating a relatively more accurate predicting ability for the prognosis of p-NENs.

**Conclusion:**

The WHO 2017 grading classification for p-NENs could successfully allocate patients into four groups with distinct clinical features and significant survival differences, which might be superior to the WHO 2010 criteria for its better prognostic stratification and more accurate predicting ability.

## Background

Pancreatic neuroendocrine neoplasms (p-NENs), namely islet cell tumors, are a group of highly heterogeneous tumors with different clinical manifestations, pathological features and long-term prognosis [1, 2]. Although p-NENs are uncommon in reported literature, data from the United States indicated that the incidence of p-NENs has increased obviously from 1.09/100,000 to 6.98/100,000 in recent decades, probably due to the development of endoscopic and radiological screening as well as the improvement of clinical awareness and diagnostic techniques for p-NENs, which has resulted in more and more attention being paid to these diseases [2–4].

Due to their pathologic heterogeneity and a spectrum of clinical behaviors of p-NENs, the criteria for predicting prognosis within uniformly classified tumors have been unsatisfactory [5]. In 2006, the European Neuroendocrine Tumor Society (ENETS) firstly proposed a separate grading system based on the cut-off point of mitotic rate per 10 high power fields (HPFs) and Ki-67 proliferative index, which defined p-NENs as Grading 1 (G1) pancreatic neuroendocrine tumors (p-NETs), G2 p-NETs and G3 pancreatic neuroendocrine carcinomas (“G3 p-NECs”) [6]. The ENETS grading scheme was later adopted in the World Health Organization (WHO) 2010 classification of neuroendocrine neoplasms because of increasing supportive evidence of its predictive power for the survival of p-NENs (Table 1) [8]. The G1/G2 p-NETs were regarded as well-differentiated in the designated ENETS/WHO grading system, while the “G3 p-NECs” were poorly-differentiated, which could present significantly different genetic, biological, treatment and survival features [9–13].

**Table 1:** Definitions of the WHO 2017 and 2010 grading classification for p-NENs and distributions of patients in the present study according to these two criteria

Definitions			Distributions
Classification	Mitotic rate^**A**^	Ki-67 proliferation index (%)^**B**^	Cases(%)
**WHO 2017 grading criteria** [7]			
Well-differentiated p-NENs:			
**NET G1**	< 2	< 3	150 (31.3%)
**NET G2**	2–20	3–20	158 (32.9%)
**NET G3**	> 20	> 20	64 (13.3%)
Poorly-differentiated p-NENs:			
** NEC G3 (small cell and large cell subtypes)**	> 20	> 20	108 (22.5%)
**WHO 2010 grading criteria** [8]			
Well-differentiated endocrine tumor, G1:			
** NET G1**	< 2	< 3	150 (31.3%)
Well-differentiated endocrine tumor, G2:			
** NET G2**	2–20	3–20	158 (32.9%)
Poorly-differentiated neoplasm: neuroendocrine carcinoma, G3 (small cell and large cell type):			
** “NEC G3”**	> 20	> 20	172 (35.8%)

**A:** The mitotic rate is based on the evaluation of mitoses in 50 high power fields in areas of higher density, and is expressed as mitoses per 10 high power fields

**B:** The Ki-67 proliferation index is based on the evaluation of ≥500 cell in areas of higher nuclear labeling (hot spot)

**Abbreviation**: *WHO* World Health Organization; *p-NENs* Pancreatic neuroendocrine neoplasms; *NET* Neuroendocrine tumors; *NEC* Neuroendocrine carcinoma; *G* Grading

In 2017, relying mainly on some established histopathologic criteria to better predict the tumor’s grade and biological behaviors, WHO officially classified p-NENs into 2 broad categories in its newly-updated grading classification (Table 1): well-differentiated p-NETs which consist of G1 p-NETs (< 2 mitoses per 10 HPFs and a Ki-67 proliferation index < 3%), G2 p-NETs (between 2 and 20 mitoses per 10 HPFs or a Ki-67 proliferation index ranging between 3 and 20%) and G3 p-NETs (> 20 mitoses per 10 HPFs or a Ki-67 proliferation index > 20% without poorly-differentiated pathological features), and poorly-differentiated p-NECs which referred to G3 p-NECs having > 20 mitoses per 10 HPFs or a Ki-67 proliferation index > 20% with poorly-differentiated small cell or large cell features [14].

The purpose of WHO 2017 grading classification for p-NENs was to improve the prediction of clinical outcomes and to determine better therapeutic strategies and patient care, which has not yet been assessed thoroughly. Whether it could better stratify p-NENs into prognostic groups and predicting their outcome has still been uncertain. In the present study, based on the relevant data from a large Chinese institution, we aimed to validate the clinical value of the WHO 2017 grading classification for p-NENs. To accomplish this, we analyzed the distribution characteristics and survival differences between each new WHO grading group. Then, we made comparisons between the WHO 2017 grading system and WHO 2010 criteria on stratifying and predicting significance for the outcome of p-NENs.

## Methods

### Patients enrollment

In the present study, we retrospectively reviewed the electronic or paper-based medical records of patients who were surgically treated and histopathologically diagnosed as p-NENs from January 2002 to December 2018 at West China Hospital of Sichuan University. We excluded patients who were only clinically suspected with related symptoms or signs but no postoperative pathological confirmation of p-NENs, as well as some patients with hereditary syndromes which were extremely rare. For included cases, we prospectively collected the relevant data such as demographic baseline, clinical presentation, imaging information, surgical procedure, perioperative outcome, etc. Our research was approved by the institutional review board of West China Hospital of Sichuan University and written informed consent was acquired on admission from all patients for their information to be used for studying purpose, which was in accordance with the general principles of the Helsinki Declaration [15].

### Tumor features

According to some recognized criteria [8, 16, 17], morphologically well-differentiated p-NENs were marked by typical neuroendocrine architectural tissues with organoid features and tumor cells with low nucleocytoplasmic ratio, abundant eosinophilic or amphophilic cytoplasm, and ovoid nuclei with salt and pepper chromatin containing well-defined nucleoli, while morphologically poorly-differentiated p-NENs were featured on nodular or solid architecture lack of organoid traits, usually with high nucleocytoplasm ratio and multifocal or extensive tumor necrosis, including small cell and large cell subtypes. For enrolled patients in the present study, all surgical specimens from tumor tissues were re-stained with hematoxylin-eosin and immunohistochemical methods, which were microscopically reviewed again by experienced pathologists in our institution. Their histopathologic analyzing results, such as morphological feature, differentiated degree, mitotic count, Ki-67 positive proliferation index, etc. were systematically documented in the prepared tabulations. After that, all p-NENs were defined into four groups of NET G1, NET G2, NET G3 and NEC G3 based on both morphological and immunohistochemical features according to their definition by WHO 2017 grading classification [14]. In terms of the tumor-node-metastasis (TNM) classification, the 8th edition of American Joint Committee on Cancer (AJCC) staging manual for p-NENs was applied respectively to different grading groups of p-NENs by combining the reports from both preoperative imaging findings, intraoperative surgical data and postoperative pathological results (in this new manual, one system was specifically proposed for G1/G2/ p-NETs, the other for “G3 p-NECs”) [18].

### Follow-up procedure

Follow-up was mainly conducted by telephone, e-mail, mail, or outpatient clinic review between July and December of 2019, leading to a median follow-up time of 40.8mon (Ranging 11.5–190.4mons). The primary outcome was overall survival (OS), which was calculated either as the time in months between the date of surgery and the date of death or last follow-up, and presented as either median survival time (MST) or estimated 5-year OS with a hazard ratio (HR) and 95% confidence intervals (CIs). Patients who were lost to follow-up were excluded in the final survival analysis models.

### Statistical analysis

Quantitative variables were reported as mean with standard deviation (SD) or median and compared using the Student’s t or the analysis of variance test. Categorical variables were presented as numbers with their frequencies as proportions (%) and compared using the Chi-square test or Fisher’s exact test. OS estimates and curves of relevant factors were generated and plotted using the Kaplan-Meier (K-M) method and compared using the log-rank test. Univariate and multivariate analysis were designed using Cox Regression proportional hazards model to validate the predicting value of the WHO 2017 grading classification for the OS of p-NENs. Difference with a two-sided *P* value less than 0.05 was considered statistically significant. All statistical analyses were carried out using IBM SPSS 25.0 statistical software.

## Results

In our present study, 150, 158, 64 and 108 patients were respectively classified into the G1/G2/G3 p-NETs and G3 p-NECs group, while 172 patients were defined as WHO 2010 “NEC G3”. The baseline demographics and tumor characteristics of p-NENs distributed by WHO 2017 and 2010 grading classification were revealed in Table 2. Comparisons of patient age (50 yrs. vs. 57 yrs., respectively, *P* = 0,034) and tumor diameter (3.5 cm vs. 5.6 cm, respectively, *P* = 0.027) between G1/G2/G3 p-NETs (i.e. well-differentiated p-NENs; *N* = 372) and G3 p-NECs (i.e. poorly-differentiated p-NENs; *N* = 108) were notably significant, while those of patient gender, functional status, incidental diagnosis, diagnosis period, tumor location, surgical margin and postoperative medical therapy present no statistical differences (*P* > 0.05). Compared with G1/G2/G3 p-NETs, G3 p-NECs exhibit more vascular infiltration (32.4% vs. 17.2%, respectively, *P* = 0.035), lymph involvement (46.3% vs. 29.3%, respectively, *P* = 0.019) and distant metastasis (29.6% vs. 16.7%, respectively, *P* = 0.041).

**Table 2:** Clinical features of p-NENs in the present study according to the WHO 2017 and 2010 grading classification

Factor	WHO 2017/2010 criteria^**A**^		WHO 2017 criteria^**A**^			WHO 2010 criteria^**A**^		
	NET G1 (***N*** = 150)	NET G2 (***N*** = 158)	NET G3 (***N*** = 64)		NEC G3 (***N*** = 108)	“NEC G3” (***N*** = 172)	All (***N*** = 480)	
Gender								
Female	88 (58.7%)	97 (61.4%)	38 (59.4%)		62 (57.5%)	100 (58.1%)	285 (59.4%)	
Age, yrs.								
Mean ± SD	45.7 ± 15.	49.1 ± 8.5	50.2 ± 7.8		54.1 ± 9.2	52.7 ± 10.	49.5 ± 8.6	
Median	2	48	52		57	253	50	
Range	45 7–68	12–82	7–71		14–80	7–80	7–82	
Tumor size, cm.	2.9 ± 2.1	5.5 ± 2.8	5.6 ± 3.5		5.9 ± 4.1	5.1 ± 3.9	5.6 ± 4.8	
Mean ± SD	2.0	4.0	4.5		5.6	5.1	4.0	
Median Range	0.3–7.2	1.2–14.5	1.8–7.6		1.6–13.1	1.6–13.1	0.3–14.5	
Functional status								
Non-functional	62 (41.3%)	88 (55.7%)	44 (68.7%)		72 (66.7%)	116 (67.4%)	266 (55.4%)	
Functional^**B**^	88 (58.7%)	70 (44.3%)	20 (31.3%)		36 (33.3%)	56 (32.6%)	214 (44.6%)	
Incidental diagnosis	35 (23.3%)	46 (29.1%)	14 (21.9%)		25 (23.1%)	39 (22.7%)	120 (25.0%)	
Diagnosis after 2010	98 (65.3%)	108 (68.4%)	54 (84.4%)		89 (82.4%)	143 (83.1%)	349 (72.7%)	
Tumor location								
Body/tail	85 (56.7%)	100 (63.3%)	43 (67.2%)		68 (62.9%)	111 (64.5%)	296 (61.7%)	
Surgical margin								
″R0^**C**^	126 (84.0%)	124 (78.5%)	33 (82.5%)		46 (70.7%)	89 (84.8%)	339 (70.6%)	
Postoperative medical therapy^**D**^	31 (20.7%)	41 (25.9%)	29 (45.3%)		61 (56.5%)	90 (52.3%)	162 (33.8%)	
Vascular infiltration	16 (10.7%)	32 (20.3%)	16 (25.0%)		35 (32.4%)	51 (29.7%)	99 (45.3%)	
Lymph involvement	35 (23.3%)	52 (32.9%)	22 (34.4%)		50 (46.3%)	72 (41.9%)	159 (33.1%)	
Distant metastasis^**E**^	20 (13.3%)	30 (19.0%)	12 (18.7%)		32 (29.6%)	44 (25.6%)	94 (19.6%)	
Hepatic	12 (8.0%)	15 (9.5%)	6 (9.4%)		20 (18.5%)	26 (15.1%)	51 (10.6%)	
Bone	5 (3.3%)	8 (5.1%)	3 (4.7%)		10 (9.3%)	13 (7.5%)	26 (5.4%)	
Lymph nodal	10 (6.6%)	14 (8.9%)	4 (6.3%)		16 (14.8%)	20 (11.6%)	44 (9.2%)	
Out of contact	30 (20.0%)	38 (24.1%)	9 (14.1%)		23 (21.3%)	32 (18.6%)	100 (20.4%)	
Dead at follow-up	44 (36.7%)	48 (40.0%)	28 (50.9%)		56 (69.1%)	84 (60.0%)	176 (46.3%)	
5-year OS	75.8%	58.4%	35.1%		11.1%	21.2%	53.0%	
MST, mons.	85.3	67.4	51.3		26.8	34.5	63.1	
TNM staging system by AJCC 2017 8th staging manual^**F**^ Stage I Stage II Stage III Stage IV	**WHO 2017 NET G1/G2/G3**^**G**^ **(*N*** **= 372)**				**WHO 2017 NEC G3**^**G**^ **(*N*** **= 108)**			
	Cases(%)	Out of contact	Dead at follow-up	5-year OS	Cases(%)	Out of contact	Dead at follow-up	3-year OS
	116 (31.2%) 108 (29.1%) 86 (23.1%) 62 (16.6%)	16 (13.8%) 20 (18.5%) 20 (23.6%) 20 (32.3%)	19 (19.0%) 23 (26.1%) 41 (62.1%) 37 (88.1%)	89.2% 70.5% 51.2% 18.9%	18 (16.7%) 30 (27.8%) 28 (25.9%) 32 (29.6%)	6 (33.7%) 10 (33.3%) 2 (7.1%) 6 (18.8%)	6 (50.0%) 13 (65.0%) 19 (73.1%) 18 (69.2%)	80.8% 33.2% 7.1% NA

**A:** The NET G1 and NET G2 were consistently defined in WHO 2017 and 2010 grading criteria for p-NENs, while the “NEC G3” of WHO 2010 criteria was composed of both NET G3 and NEC G3 of WHO 2017 criteria [8, 14]

**B:** Referring to insulinoma, gastrinoma, vasoactive intestinal polypeptidoma, adrenocorticotropic hormone adenoma, glucagonoma, pheochromocytoma, etc.

**C:** Referring to radical resections with both grossly and microscopically negative surgical margins

**D:** Referring to conventional chemotherapy and novel molecular targeted therapies

**E:** Distant metastases (hepatic, bone, lymph nodal) for patients with p-NENs was present at diagnosis

**F:** In the AJCC 2017 staging manual, one TNM system was originally proposed for G1/G2 p-NETs, the other for “G3 p-NECs” of WHO 2010 criteria

**G:** The G3 p-NETs of WHO 2017 grading classification were also staged by the system for G1/G2 p-NETs, while the G3 p-NECs of WHO 2017 criteria were staged by the system for pancreatic exocrine adenocarcinomas, as we have demonstrated in the previous report [19]

**Abbreviations**: *p-NENs* Pancreatic neuroendocrine neoplasms; *WHO* World Health Organization; *NET* Neuroendocrine tumors; *NEC* Neuroendocrine carcinoma; *G* Grading; *TNM* Tumor-node-metastasis; *AJCC* American Joint Committee On Cancer; *NA* Not applicable; *OS* Overall survival; *MST* Median survival time

**Fig. 1:**
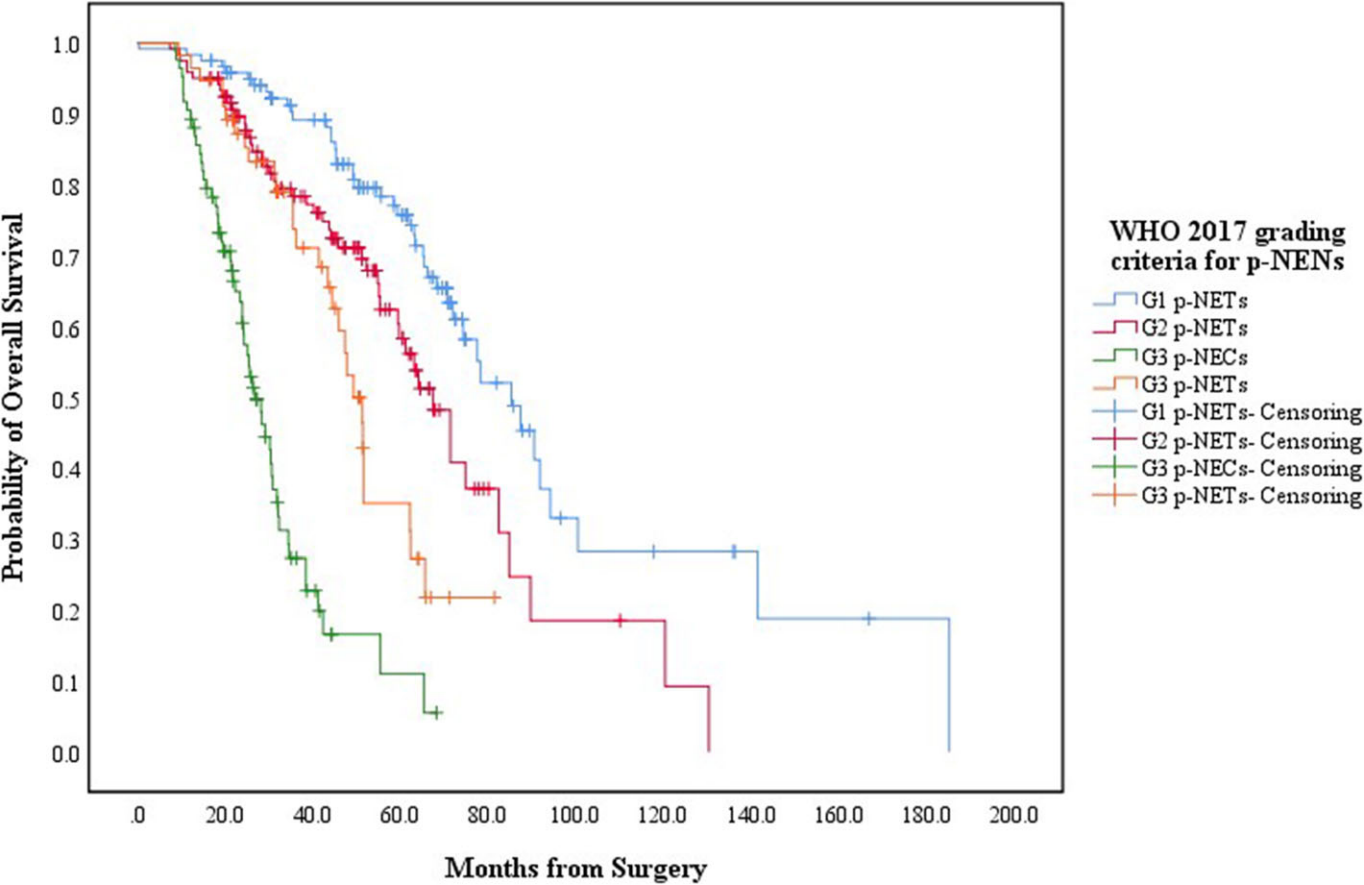
Kaplan-Meier estimates for the OS of p-NENs, according to the WHO 2017 grading classification

**Fig. 2:**
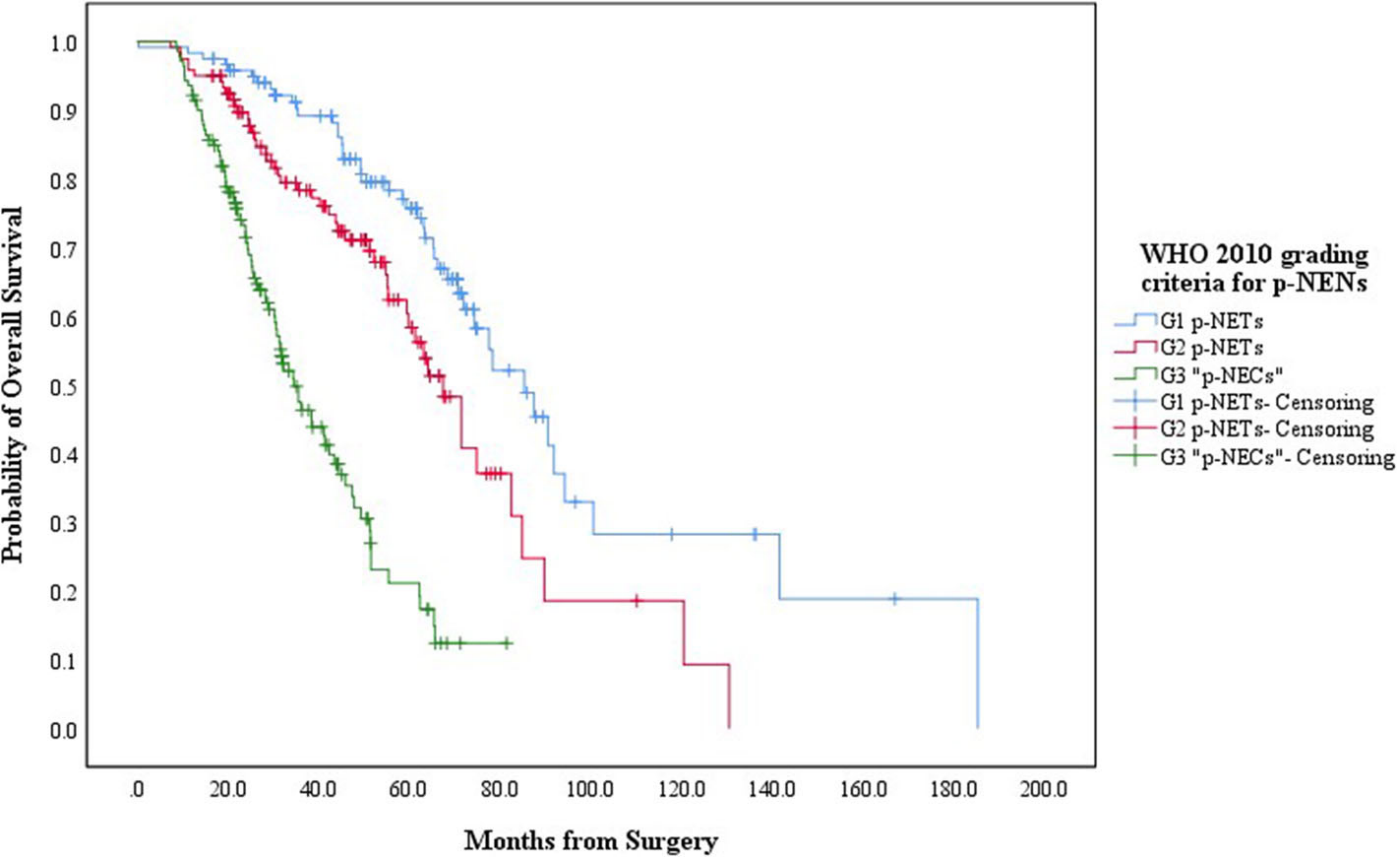
Kaplan-Meier estimates for the OS of p-NENs, according to the WHO 2010 grading classification

When the follow-up ended, there were 120 patients with G1 p-NETs, 120 with G2 p-NETs, 55 with G3 p-NETs and 85 with G3 p-NECs in touch, in which 44, 48, 28 and 56 patients respectively died due to the progression of p-NENs (Table 2). The 5-year OS for patients with G1/G2/G3 p-NETs and G3 p-NECs was 75.8, 58.4, 35.1 and 11.1%, respectively, with a MST of 85.3mons (95% CIs: 72.2–98.4), 67.4mons (95% CIs: 58.7–76.1), 51.3mons (95% CIs: 46.3–56.3) and 26.8mons (95% CIs: 22.8–30.8), while that for patients with WHO 2010 “G3 p-NECs” was 21.2%, with a MST of 34.5mon (95% CIs: 28.9–40.1). Patients with G1 p-NETs by WHO 2017 or 2010 criteria present notably better survival than those with G2 p-NETs (*P* = 0.03, Fig. 1; *P* = 0.03, Fig. 2; respectively). Survival of the WHO 2017 G3 p-NETs were significantly worse than that of G1 p-NETs (*P* < 0.001) or G2 p-NETs (*P* = 0.023; Fig. 1), as well as that when comparing G3 p-NECs with G1 p-NETs (*P* < 0.001) or G2 p-NETs (*P* < 0.001; Fig. 1), while patients with G3 p-NECs also showed statistically shorter survival than those with G3 p-NETs (*P* < 0.001; Fig. 1). Patients with WHO 2010 “G3 p-NECs” present worse survival than those with G1 p-NETs (*P* < 0.001) or G2 p-NETs (*P* < 0.001; Fig. 2) as well.

**Fig. 3:**
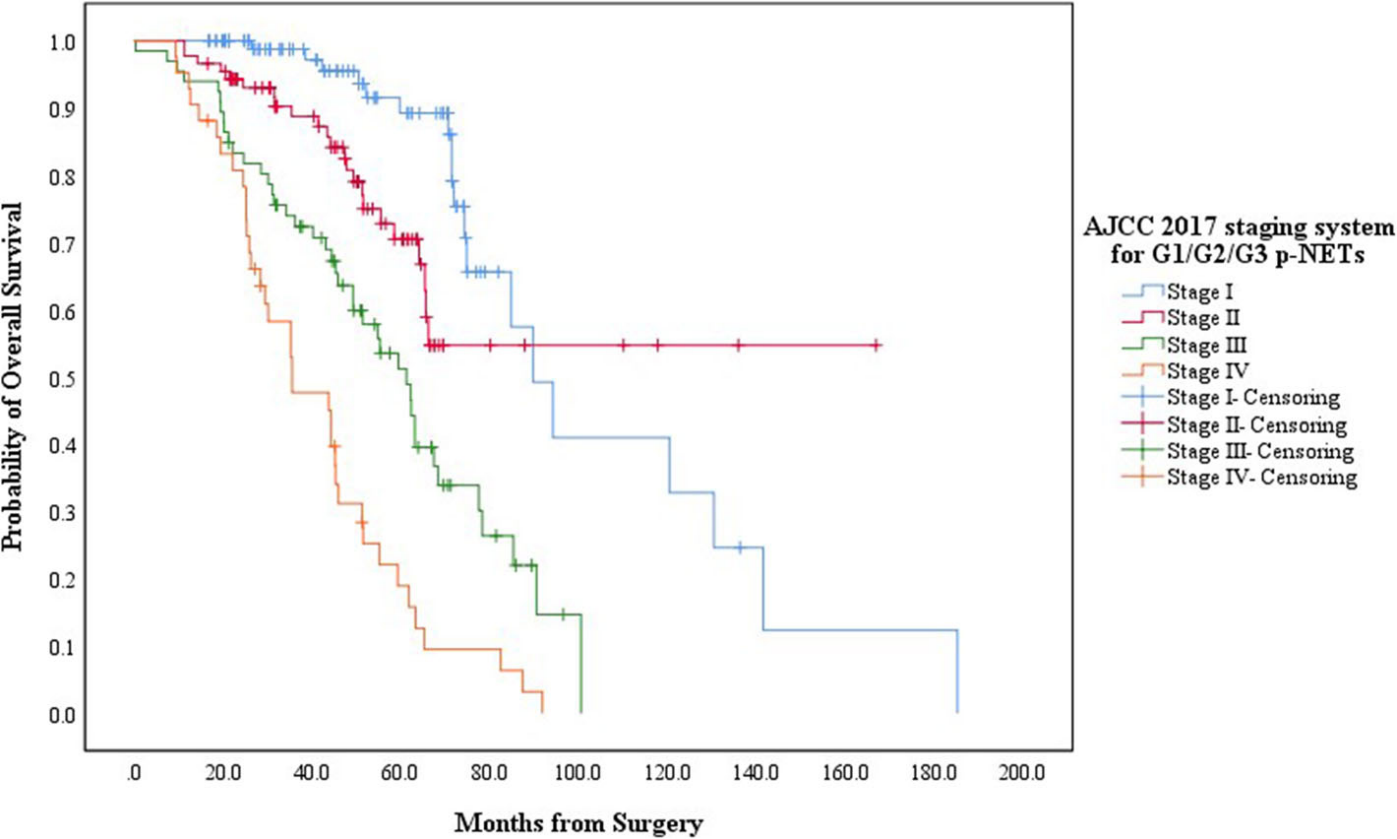
Kaplan-Meier estimates for the OS of G1/G2/G3 p-NETs, according to the AJCC 2017 staging system originally proposed for G1/G2 p-NETs

**Fig. 4:**
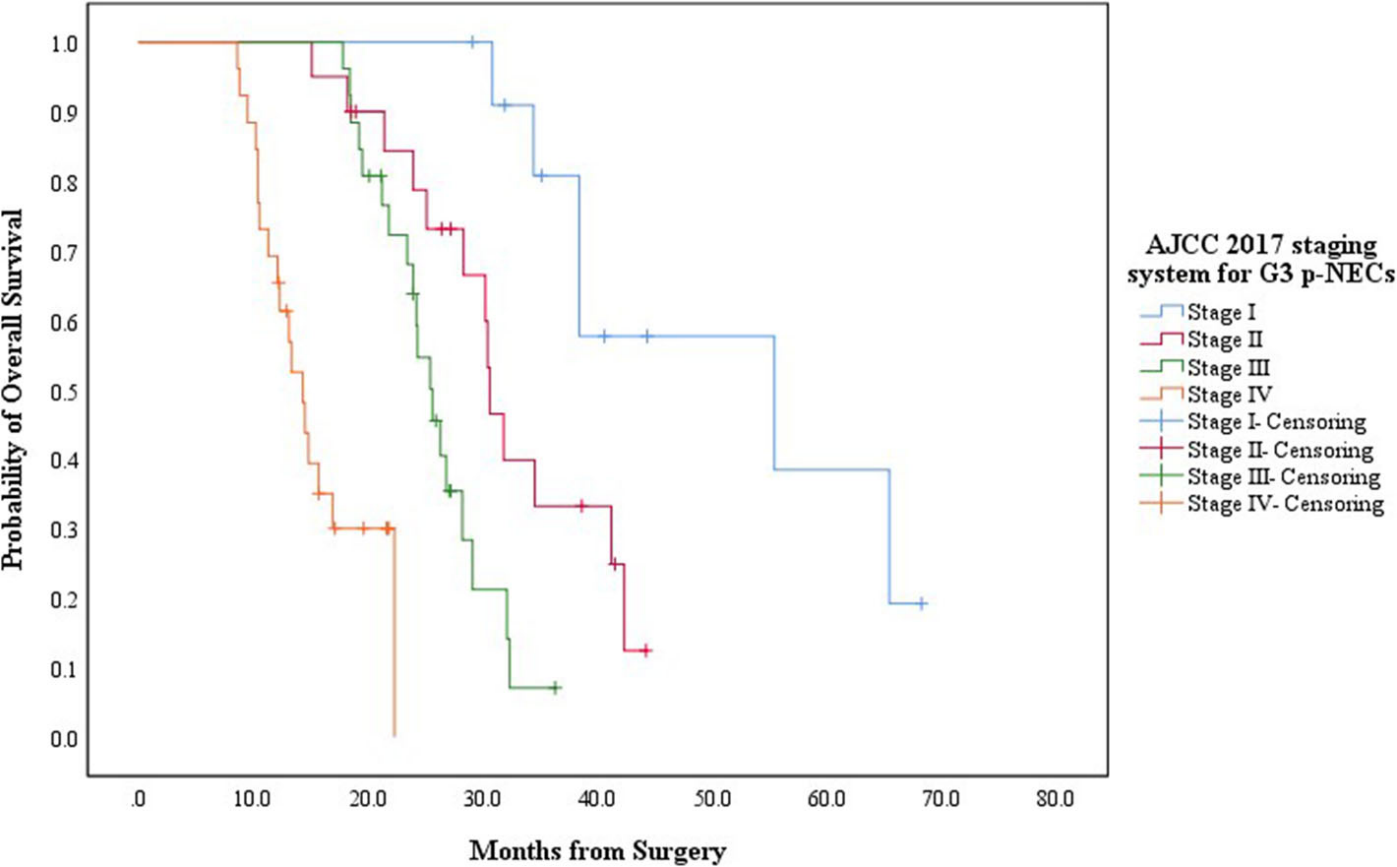
Kaplan-Meier estimates for the OS of G3 p-NECs, according to the AJCC 2017 staging system originally proposed for “G3 p-NECs”

The diverse clinicopathological features of each new grading group of p-NENs has led to significant different distributions of tumor stage (Table 2). For G1/G2/G3 p-NETs, by applying one AJCC 2017 TNM staging system which was originally proposed for G1/G2 p-NETs, there were respectively 116, 108, 86 and 62 patients defined in Stage I, Stage II, Stage III and Stage IV. The estimated 5-year OS of patients in each new stage was 89.2, 70.5, 51.2 and 18.9%, respectively, with a MST of 89.7mons (95% CIs: 76.1–103.3), not reached, 61.2mons (95% CIs: 52.5–69.9) and 35.5mons (95% CIs: 19.8–51.5). Patients in Stage I or Stage II both showed notably longer survivals than those in Stage III (*P* < 0.001, *P* < 0.001; respectively) or Stage IV (*P* = 0.001, *P* < 0.001; respectively; Fig. 3). Moreover, there were also significant survival differences when comparing Stage I with Stage II (*P* = 0.037) or Stage III with Stage IV (*P* = 0.001, Fig. 3). For G3 p-NECs, by using the other new AJCC staging system which was primarily suggested for “G3 p-NECs”, 18, 30, 28 and 32patients were respectively distributed from Stage I to Stage IV. The calculated 3-year OS for patients in each new stage was 80.8, 33.2, 7.1% and NA, respectively, with a MST of 55.4mons (95% CIs: 22.3–88.5), 30.6mons (95% CIs: 28.6–32.6), 25.6mons (95% CIs: 23.3–27.9) and 14.3mons (95% CIs: 12.2–16.4). Patients in Stage I or Stage II present statistically better survivals than those in Stage III (*P* < 0.001, *P* < 0.001; respectively) or Stage IV (*P* = 0.023, *P* < 0.001; respectively; Fig. 4). Meanwhile, survival differences when comparing Stage I with Stage II (*P* = 0.014) or Stage III with Stage IV (*P* < 0.001; Fig. 4) were also significant.

Using Cox Regression proportional hazards models, we performed univariate and multivariate analysis to evaluate the predicting value of certain factors with the OS of p-NENs (Table 3). According to our demonstrations, patient gender and age, tumor location and incidental diagnosis weren’t statistically significant in univariate analysis (*P* > 0.05), while functional status, tumor diameter and postoperative medical therapy were not significant in multivariate analysis (P > 0.05). Radical resection, vascular infiltration, lymph involvement, distant metastasis and grading by WHO 2017 and 2010 criteria were statistically significant in both univariate (*P* < 0.05) and multivariate analysis (*P* < 0.05). Our analysis revealed that both WHO 2017 and 2010 grading criteria could be independent predictor for the OS of p-NENs (*P* = 0.016, *P* = 0.022; respectively). The 95% CIs of WHO 2010 grading classification (0.201–13.374) was slightly larger than that of WHO 2017 criteria (0.983–9.454), indicating a relatively inaccurate predicting ability.

**Table 3:** Univariate and multivariate analysis of factors with the OS of p-NENs using Cox Regression proportional hazard models

Factor	Univariate Analysis	WHO 2017 criteria Multivariate Analysis^**A**^	WHO 2010 criteria Multivariate Analysis^**A**^
	HR(95%CIs) P	HR(95%CIs) P	HR(95%CIs) P
Gender			
Male^**B**^			
Female	0.953 (0.467–3.252) 0.537		
Age, yr.			
< Median			
≥ Median	1.211 (0.587–1.256) 0.079		
Tumor location			
Head			
Body/tail	1.436 (0.912–2.579) 0.408		
Functional status			
Functional			
Non-functional	1.725 (0.436–3.926) **0.027**	0.894 (0.583–1.457) 0.635	0.616 (0.589–1.368) 0.441
Incidental diagnosis			
No			
Yes	0.984 (0.457–1.562) 0.336		
Radical resection			
Yes			
No	4.113 (0.384–7.335) **< 0.001**	0.993 (0.309–1.873) **0.009**	1.023 (0.574–2.359) **0.015**
Postoperative medical therapy			
Yes			
No	1.518 (0.366–2.735) **0.045**	0.845 (0.673–1.419) 0.357	0.903 (0.449–1.525) 0.745
Tumor diameter			
< Median			
≥ Median	1.005 (0.567–2.138) **0.024**	0.665 (0.357–1.983) 0.341	0.883 (0.436–1.843) 0.452
Vascular infiltration			
No			
Yes	2.138 (0.543–4.113) **0.037**	1.255 (0.843–2.059) **0.012**	0.993 (0.519–1.725) **0.039**
Lymph involvement			
No			
Yes	3.542 (0.343–6.25) **0.008**	2.357 (0.331–5.369) **0.036**	1.924 (0.536–3.454) **0.025**
Distant metastasis			
No			
Yes	5.112 (0.478–10.205) **< 0.001**	4.124 (0.385–8.359) **0.027**	2.445 (0.501–7.374) **0.048**
Grading by WHO 2010 criteria			
NET G1/G2			
“NEC G3”	4.425 (0.454–14.346) **< 0.001**	NA	2.445 (0.201–13.374) **0.022**
Grading by WHO 2017 criteria			
NET G1/G2/G3			
NEC G3	6.634 (0.634–8.257) **< 0.001**	4.562 (0.983–9.454) **0.016**	NA

**A:** Predicting value of the WHO 2017 and 2010 grading classification for the OS of p-NENs was built and evaluated in separate Regression proportional hazard models

**B:** The above one of related factor was regarded as a reference in Cox analysis

**Abbreviation**: *OS* Overall survival; *p-NENs* Pancreatic neuroendocrine neoplasms; *WHO* World Health Organization; *HR* Hazard ratio; *CIs* Confidence intervals; *NET* Neuroendocrine tumors; *NEC* Neuroendocrine carcinoma; *G* Grading; *NA* Not applicable

## Discussion

A uniform classification for p-NENs has been lacking to stratify p-NENs into prognostic groups, although several varying systems have been devised, analyzed, and compared for p-NENs [20, 21]. In 2010, the WHO grading system distinguished G1 p-NETs from G2 p-NETs and “G3 p-NECs” based on mitotic rate and Ki-67 proliferative index [6, 8], which has been proven to be prognostic for the OS of p-NENs [9–13]. Although the WHO 2010 grading classification for p-NENs represented an important step toward adopting a uniform grading system with widespread acceptance, its weakness appeared gradually.

Firstly, WHO suggested the higher of the two parameters be used to assign the final grade (typically, the Ki-67 index often pointed to the higher WHO grade) when mitotic rate and Ki-67 index were sometimes discordant [8]. This would inevitably increase the number of cases of “G3 p-NECs”, which was demonstrated by Basturk et al. that mitotic G2/Ki-67 “G3 p-NECs” biologically behaved more like mitotic G2/Ki-67 G2 p-NETs [22]. They found that p-NENs with a Ki-67 proliferative index > 20%, if well-differentiated, were more aggressive than G2 but significantly less aggressive than “G3 p-NECs” with poorly differentiated features (large or small cell type) [22]. Furthermore, the WHO 2010 grading classification just used the terminology “high-grade” and “poorly-differentiated” interchangeably for neoplasms in the G3 category, while recent studies have further focused on the heterogeneity of “G3 p-NECs”, in which some might primarily present a high Ki-67 proliferative rate but be morphologically well-differentiated [23]. Sorbye et al. demonstrated the WHO 2010 “G3 p-NECs” were morphologically and biologically heterogenous, in which they reported a lower response rate after platinum-based systemic chemotherapy (15% vs. 42%, respectively; *P* < 0.05), but a longer MST (14mon vs. 10mon, respectively; *P* < 0.05) among tumors with a Ki-67 < 55%, compared with those having a higher Ki-67 index [7]. Similar conclusions have also been reached that G3 p-NENs might consist of two distinct subgroups: well-differentiated p-NETs with a high proliferative rate (grade-discordant G2 p-NETs or morphologically G3 p-NETs) and true poorly-differentiated p-NECs (small-cell or large-cell G3 p-NECs) [24–26].

The previous work eventually formed the basis for the WHO grading classification published in 2017 (Table 1), which officially defined p-NENs into two broad categories (well-differentiated and poorly-differentiated) and four groups (NET G1/G2/G3 and NEC G3) in the light of both morphological differentiation and grading upon proliferation rate [14]. However, this new system has not yet been validated. According to the comprehensive analysis of p-NENs in the present study, we revealed three major findings. First, the WHO 2017 grading classification could well distribute p-NENs into four significant groups with different clinical features and long-term survivals. Second, the new WHO system was superior to WHO 2010 criteria for better stratifying ability and more accurate predicting ability for the OS of p-NENs. Finally, patients with different WHO 2017 grading p-NENs could be well staged by the new AJCC 8th TNM staging manual.

According to the definitions of WHO 2017 and 2010 grading classification for p-NENs, their main difference was that the WHO 2010 “G3 p-NECs” group was now divided into WHO 2017 G3 p-NET and G3 p-NECs (Table 1). We have just reported in one study that comparisons of patient demographics and tumor characteristics of G3 p-NETs and G3 p-NECs weren’t significant (*P* > 0.05), although the tumor diameter of G3 p-NETs seemed be smaller than that of G3 p-NECs (4.5 cm vs. 5.6 cm, respectively; *P* = 0.059) [27]. Hereby, in Table 2, comprehensive comparisons were made for related factors between well-differentiated neoplasms (i.e. G1/G2/G3 p-NETs) and poorly-differentiated ones (i.e. G3 p-NECs). We found that the patient age of G1/G2/G3 p-NETs was notably younger than that of G3 p-NECs (3.5 cm vs. 5.6 cm, respectively; *P* = 0.027) and the tumor diameter of G1/G2/G3 p-NETs was statistically smaller than that of G3 p-NECs (50 yrs. vs. 57 yrs., respectively; *P* = 0.034). Meanwhile, compared with G1/G2/G3 p-NETs, G3 p-NECs present significantly more vascular infiltration (32.4% vs. 17.2%, respectively; *P* = 0.035), lymph involvement (46.3% vs. 29.3%, respectively; *P* = 0.019) and distant metastasis (29.6% vs. 16.7%, respectively; *P* = 0.041). Referring to the results above [27], statistical differences of these clinicopathological features might be caused by the integration of G1/G2/G3 neoplasms, forming the category of well-differentiated p-NENs, as McCall et al. have demonstrated in their study [28].

G1/G2/G3 p-NETs were usually slow-growing tumors with equal sex preference occurring over a broad age range, highest incidence peak between third and sixth decade, while G3 p-NECs had an incidence peak in the sixth to seventh decade, whose clinical presentation was very similar to pancreatic exocrine adenocarcinomas (p-EACs) [17]. Our analysis indicated that patient gender among each new grading group had a slight female predominance with a peak median incidence age ranging from 45 yrs. to 57 yrs. and that p-NENs more frequently involved the body or tail of pancreas (Table 2). In terms of the survival of p-NENs, the WHO 2017 and 2010 grading classification both showed significantly decreased survivals as grade increased (Fig. 1, Fig. 2; respectively). Most importantly, the estimated 5-year OS of G3 p-NETs was statistically better than that of G3 p-NECs (35.1% vs. 11.1%, respectively; *P* < 0.001) but notably worse than that of G2 p-NETs (35.1% vs. 58.4%, respectively; *P* = 0.023) and G1 p-NETs (35.1% vs. 75.8%, respectively; *P* < 0.001; Fig. 1). This situation was in agreement with the reported results we mentioned above [25–27]. We then revealed that although the WHO 2017 and 2010 criteria could be independent predictor for the OS of p-NENs (*P* = 0.016, *P* = 0.022, respectively; Table 3), the 95% CIs of WHO 2017 grading classification (0.983–9.454) was slightly smaller than that of WHO 2010 criteria (0.201–13.374), indicating a relatively better predicting accuracy.

Another concern of our analysis was the TNM staging classification for p-NENs. In 2010, AJCC began to apply its TNM staging system to p-NENs [19], which derived from the staging algorithm for p-EACs and was proven to be convenient but a little oversimplified for p-NENs [29, 30]. In 2017, AJCC updated its staging manual for p-NENs (i.e. 8th edition), in which AJCC highlighted that the novel system for p-NENs should only be applied to G1/G2 p-NETs, while “G3 p-NECs” be staged by the revised one for p-EACs [18]. The two new independent AJCC staging systems for p-NENs have been separately demonstrated to be superior to the AJCC 7th edition system in two previous studies [31, 32]. Recently, considering the heterogeneity with “G3 p-NECs”, we for the first time attempted to evaluate which new system G3 p-NETs should be better staged by [27]. We concluded that the AJCC 8th staging systems introduced for G1/G2 p-NETs and “G3 p-NECs” were both practical for G3 p-NETs, while the one originally applied to G1/G2 p-NETs appeared to be superior in performance [27]. Therefore, in the present study, we firstly staged G1/G2/G3 p-NETs together by one new AJCC system for G1/G2 p-NETs and staged G3 p-NECs by the other one for “G3 p-NECs” (Table 2). According to our analysis, both G1/G2/G3 p-NETs and G3 p-NECs could be well classified into four prognostic groups by their corresponding AJCC system, respectively, with statistically different stage distributions on their OS (*P* < 0.05; Fig. 3 and Fig. 4).

Our study had some limitations. First of all, it was also a retrospective study in which data analysis and patient’s recruitment were over a long duration. Secondly, the accumulative OS was estimated by K-M methods due to some cases with a short follow-up time. Then, our analysis derived from one single medical institution which might reduce the statistical power between factors and survival outcomes. Finally, all patients had surgically-resected disease and applicability to patients presenting with advanced disease needs to be validated. Hereby, a particular implication for patients with G3 p-NECs, particularly those with metastatic disease at presentation, given that surgery would not be considered as standard management for most patients with G3 p-NECs [33–35]. Therefore, a prospectively designed study from multi centers and with a long follow-up time is still needed to confirm our results.

## Conclusion

In a word, we concluded that the WHO 2017 grading classification for p-NENs could successfully allocate patients into four groups with distinct clinical features and significant survival differences, which might be superior to the WHO 2010 criteria for its better prognostic stratification and more accurate predicting ability. Our demonstration supported the wide use of WHO 2017 grading classification to p-NENs in current clinical practice.

## Data Availability

The data and materials of our present research couldn’t be shared at this time as the data and materials also formed part of an ongoing study, while they could be available in the near future from the corresponding author upon request after the accomplishment of our ongoing study.

## Abbreviations

P-NENs: Pancreatic neuroendocrine neoplasms
ENETS: European neuroendocrine tumor society
HPFs: High power fields
G: Grading
p-NETs: Pancreatic neuroendocrine tumors
G3 p-NECs: Pancreatic neuroendocrine carcinomas
WHO: World health organization
TNM: Tumor-node-metastasis
AJCC: American joint committee on cancer
MST: Median survival time
HR: Hazard ratio
CIs: Confidence intervals
SD: Standard deviation
K-M: Kaplan-meier
p-EACs: Pancreatic exocrine adenocarcinomas.
